# Supplementary material for: Modeling glioblastoma heterogeneity as a dynamic network of cell states
Source: Mol Syst Biol. 2021 Sep 16;17(9):e10105. doi: 10.15252/msb.202010105 (PMC8444284; doi:10.15252/msb.202010105)
Supplement: Supplementary file 5 — Source Data for Figure 3 [file MSB-17-e10105-s001.zip › Figure3A_sourcedata/GSEA_3065/hallmarks_state6.GseaPreranked.1623416308702/HALLMARK_COAGULATION.html]

Details for gene set HALLMARK\_COAGULATION[GSEA]

|  || Dataset | state6 |
| Phenotype | NoPhenotypeAvailable |
| Upregulated in class | na\_neg |
| GeneSet | HALLMARK\_COAGULATION |
| Enrichment Score (ES) | -0.5049705 |
| Normalized Enrichment Score (NES) | -1.8075535 |
| Nominal p-value | 0.0 |
| FDR q-value | 0.004085336 |
| FWER p-Value | 0.025 |
Table: GSEA Results Summary

  

Fig 1: Enrichment plot: HALLMARK\_COAGULATION      
 Profile of the Running ES Score & Positions of GeneSet Members on the Rank Ordered List

  

| PROBE | GENE SYMBOL | GENE\_TITLE | RANK IN GENE LIST | RANK METRIC SCORE | RUNNING ES | CORE ENRICHMENT || 1 | MSRB2 |  |  | 119 | 0.204 | 0.0298 | No |
| 2 | DUSP14 |  |  | 592 | 0.115 | 0.0003 | No |
| 3 | PEF1 |  |  | 754 | 0.102 | 0.0035 | No |
| 4 | RAC1 |  |  | 816 | 0.097 | 0.0172 | No |
| 5 | FYN |  |  | 817 | 0.097 | 0.0378 | No |
| 6 | CRIP2 |  |  | 1052 | 0.083 | 0.0286 | No |
| 7 | ISCU |  |  | 1241 | 0.074 | 0.0229 | No |
| 8 | CAPN2 |  |  | 1385 | 0.068 | 0.0210 | No |
| 9 | SIRT2 |  |  | 1955 | 0.049 | -0.0336 | No |
| 10 | GNG12 |  |  | 2317 | 0.038 | -0.0668 | No |
| 11 | FN1 |  |  | 2839 | 0.026 | -0.1209 | No |
| 12 | USP11 |  |  | 3393 | 0.013 | -0.1814 | No |
| 13 | RABIF |  |  | 3769 | 0.005 | -0.2231 | No |
| 14 | SERPINE1 |  |  | 3811 | 0.004 | -0.2269 | No |
| 15 | WDR1 |  |  | 4013 | 0.000 | -0.2498 | No |
| 16 | GNB2 |  |  | 4215 | -0.003 | -0.2720 | No |
| 17 | S100A13 |  |  | 4664 | -0.012 | -0.3207 | No |
| 18 | ARF4 |  |  | 4760 | -0.014 | -0.3287 | No |
| 19 | CPQ |  |  | 4765 | -0.014 | -0.3263 | No |
| 20 | MMP15 |  |  | 4887 | -0.016 | -0.3366 | No |
| 21 | FURIN |  |  | 4962 | -0.017 | -0.3414 | No |
| 22 | LGMN |  |  | 4983 | -0.018 | -0.3399 | No |
| 23 | TIMP3 |  |  | 5017 | -0.019 | -0.3397 | No |
| 24 | PLAU |  |  | 5312 | -0.025 | -0.3681 | No |
| 25 | CTSV |  |  | 5544 | -0.029 | -0.3882 | No |
| 26 | THBS1 |  |  | 5670 | -0.032 | -0.3957 | No |
| 27 | CAPN5 |  |  | 5758 | -0.034 | -0.3985 | No |
| 28 | CSRP1 |  |  | 5955 | -0.037 | -0.4130 | No |
| 29 | PROS1 |  |  | 6263 | -0.044 | -0.4388 | No |
| 30 | C1R |  |  | 6276 | -0.044 | -0.4308 | No |
| 31 | LTA4H |  |  | 6318 | -0.045 | -0.4259 | No |
| 32 | SH2B2 |  |  | 6396 | -0.047 | -0.4247 | No |
| 33 | MMP11 |  |  | 6442 | -0.048 | -0.4196 | No |
| 34 | CTSO |  |  | 6552 | -0.052 | -0.4211 | No |
| 35 | BMP1 |  |  | 6662 | -0.054 | -0.4219 | No |
| 36 | PREP |  |  | 6667 | -0.055 | -0.4108 | No |
| 37 | MMP2 |  |  | 7016 | -0.065 | -0.4367 | No |
| 38 | DUSP6 |  |  | 7215 | -0.071 | -0.4441 | No |
| 39 | LRP1 |  |  | 7748 | -0.093 | -0.4852 | Yes |
| 40 | FBN1 |  |  | 7777 | -0.094 | -0.4683 | Yes |
| 41 | CTSB |  |  | 7870 | -0.099 | -0.4577 | Yes |
| 42 | HTRA1 |  |  | 8245 | -0.126 | -0.4737 | Yes |
| 43 | SPARC |  |  | 8303 | -0.132 | -0.4520 | Yes |
| 44 | GSN |  |  | 8339 | -0.137 | -0.4269 | Yes |
| 45 | CLU |  |  | 8387 | -0.145 | -0.4014 | Yes |
| 46 | LAMP2 |  |  | 8425 | -0.149 | -0.3739 | Yes |
| 47 | ANXA1 |  |  | 8651 | -0.213 | -0.3542 | Yes |
| 48 | PRSS23 |  |  | 8688 | -0.233 | -0.3087 | Yes |
| 49 | TIMP1 |  |  | 8751 | -0.290 | -0.2541 | Yes |
| 50 | TFPI2 |  |  | 8752 | -0.290 | -0.1924 | Yes |
| 51 | ADAM9 |  |  | 8756 | -0.296 | -0.1298 | Yes |
| 52 | CD9 |  |  | 8762 | -0.303 | -0.0658 | Yes |
| 53 | PLAT |  |  | 8772 | -0.327 | 0.0027 | Yes |
Table: GSEA details [plain text format]

  

Fig 2: HALLMARK\_COAGULATION: Random ES distribution      
 Gene set null distribution of ES for **HALLMARK\_COAGULATION**
